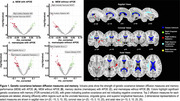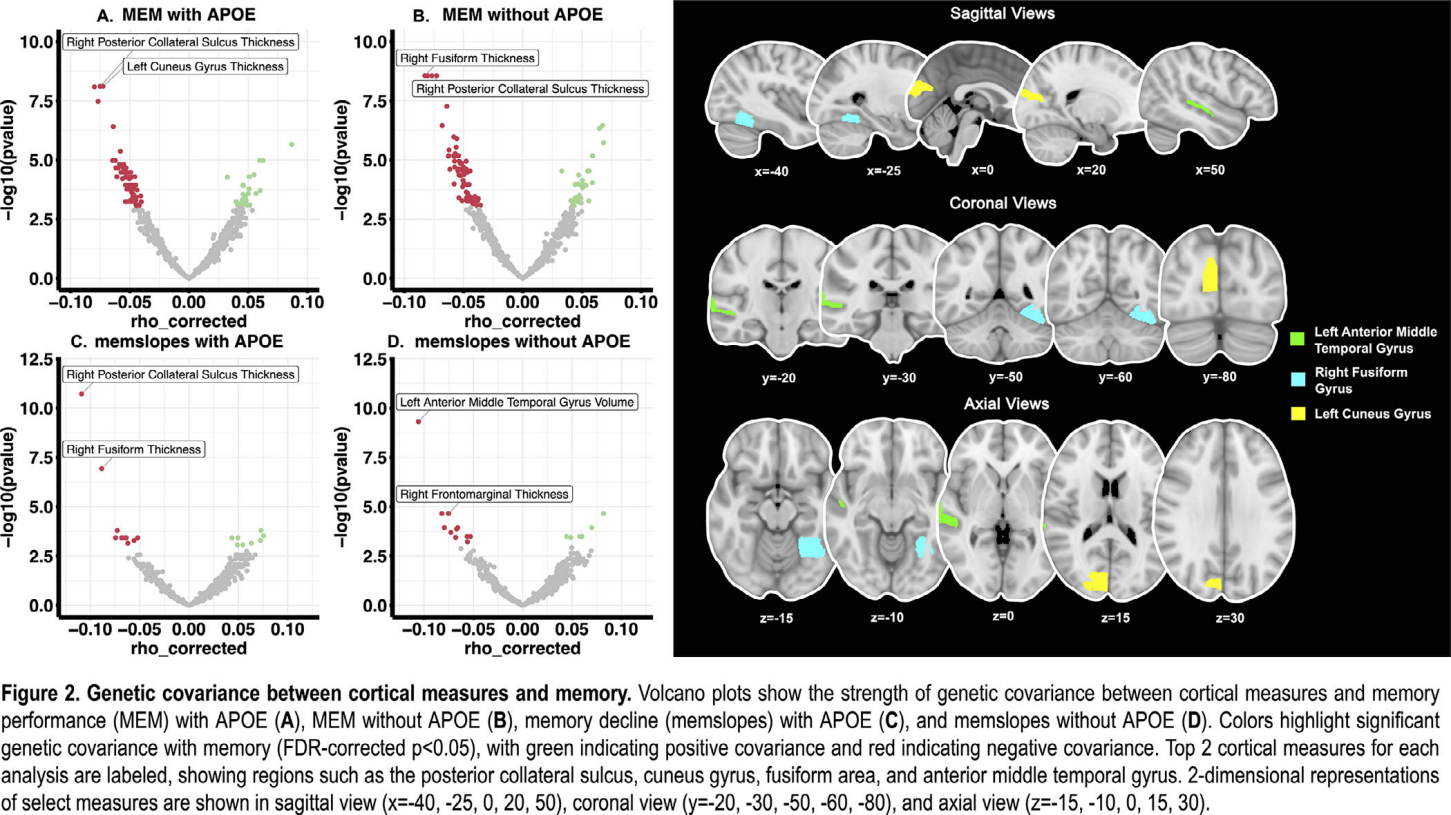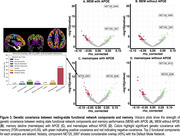# Exploring the Genetic Correlation Between Memory Performance and Multimodal Neuroimaging Phenotypes

**DOI:** 10.1002/alz70856_104003

**Published:** 2025-12-26

**Authors:** Yisu Yang, Aditi Sathe, Kurt Schilling, Leslie S. Gaynor, Seo‐Eun Choi, Brandon Klinedinst, Michael L. Lee, Emily H. Trittschuh, Elizabeth Sanders, Logan Dumitrescu, Bennett A. Landman, Paul K Crane, Timothy J. Hohman, Derek B. Archer

**Affiliations:** ^1^ Vanderbilt Memory & Alzheimer's Center, Vanderbilt University Medical Center, Nashville, TN, USA; ^2^ Vanderbilt Memory and Alzheimer's Center, Vanderbilt University School of Medicine, Nashville, TN, USA; ^3^ Vanderbilt University Institute of Imaging Science, Vanderbilt University Medical Center, Nashville, TN, USA; ^4^ Department of Radiology and Radiological Sciences, Vanderbilt University Medical Center, Nashville, TN, USA; ^5^ Division of Geriatric Medicine, Department of Medicine, Vanderbilt University Medical Center, Nashville, TN, USA; ^6^ Vanderbilt University Medical Center, Nashville, TN, USA; ^7^ Department of Medicine, University of Washington, Seattle, WA, USA; ^8^ Department of Psychiatry and Behavioral Sciences, University of Washington School of Medicine, Seattle, WA, USA; ^9^ VA Puget Sound Health Care System, Seattle, WA, USA; ^10^ Vanderbilt Genetics Institute, Vanderbilt University Medical Center, Nashville, TN, USA; ^11^ Department of Biomedical Engineering, Vanderbilt University, Nashville, TN, USA; ^12^ Department of Electrical and Computer Engineering, Vanderbilt University, Nashville, TN, USA; ^13^ Department of General Internal Medicine, University of Washington School of Medicine, Seattle, WA, USA

## Abstract

**Background:**

Memory performance is a strong endophenotype for Alzheimer's disease (AD). While the neuroimaging correlates of memory have been explored, their underlying genetic relationship remains unclear.

**Method:**

We leveraged GeNetic cOVariance Analyzer (GNOVA) to estimate genetic covariance between two sets of summary statistics from large‐scale genome‐wide association studies (GWAS). The first was drawn from Elliot et al. (2018)'s GWAS on 3,143 imaging‐derived phenotypes (IDP) in 8,428 participants of the UK Biobank; IDPs included 675 diffusion‐weighted MRI (dMRI) measures, 675 T1‐weighted MRI measures, and 1793 resting‐state functional MRI (rs‐fMRI) network components derived from group independent component analysis. The second was drawn from a previous GWAS by our group, conducted on memory performance (24,216 participants) and decline (114,070 cognitive sessions) leveraging harmonized cognitive data from 4 longitudinal cohorts of aging: ACT, ADNI, NACC, and ROSMAP. GNOVA was run on cross‐sectional memory performance and longitudinal memory decline, with follow‐ups conducted after removing *APOE* region, totaling 4 analyses. GNOVA results were stratified by imaging modality to determine the top IDPs exhibiting greatest genetic covariance with memory performance and decline for dMRI, T1‐weighted MRI, and rs‐fMRI features, respectively. Percent overlap in volume with Schaefer (2018)'s 7‐network atlas was computed for each top rs‐fMRI feature.

**Result:**

Top dMRI regions exhibiting greatest genetic covariance with memory included the uncinate fasciculus, cingulate gyrus, and superior longitudinal fasciculus (Figure 1). Top T1 regions included posterior collateral sulcus, cuneus gyrus, fusiform gyrus, and anterior middle temporal gyrus (Figure 2). Among top resting‐state functional network components, NET25_0067 and NET100_0046 showed considerable overlap with the Default Mode Network (45% and 34%, respectively), whereas NET100_0013 showed large overlap with the Visual Network (52%). An illustration of NET25_0067 is shown in Figure 3.

**Conclusion:**

Genetic covariance analyses between memory and multimodal IDPs displayed wide‐ranging significant results, with the most pronounced effects centered within medial temporal lobe and adjacent structures. These associations largely parallel AD brain vulnerability, particularly within regions associated with the default mode network. Furthermore, the identification of brain traits beyond the hippocampus as associated with the genetic architecture of memory suggests additional neural correlates to evaluate for future biomarker development.